# *catena*-Poly[[[pyridinecopper(II)]-(μ-2-oxidonaphthalene-1-carbaldehyde pico­linoylhydrazonato)-[pyridinecopper(II)]-μ-sulfato] diethyl ether hemisolvate]

**DOI:** 10.1107/S1600536809012653

**Published:** 2009-04-18

**Authors:** Quanchang Wang, Dacheng Li, Daqi Wang

**Affiliations:** a College of Chemistry and Chemical Engineering, Liaocheng University, Shandong 252059, People’s Republic of China

## Abstract

The title compound, {[Cu_2_(C_17_H_11_N_3_O)(SO_4_)(C_5_H_5_N)_2_]·0.5C_4_H_10_O}_*n*_, was syn­thesized by the reaction of 2-hydr­oxy-1-naphthyl­aldehyde-2-pyridine­carboxyl­hydrazone with copper sulfonate. A one-dimensional polymer was obtained *via* self-assembly. Each Cu ion is located in a distorted square-pyramidal coordination environment, with one Cu ion coordinated by two N and three O atoms, while the other links to two O and three N atoms. In the crystal, weak inter­molecular C—H⋯O inter­actions connect the chains into a two-dimensional network.

## Related literature

For the biological activity of aroylhydrazones, see Armstrong *et al.* (2003 ▶). For the crystal structure of a copper complex with a related picolinoylhydrazone derivative, see: Bai *et al.* (2006 ▶).
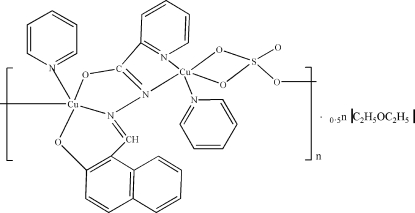


## Experimental

### 

#### Crystal data

[Cu_2_(C_17_H_11_N_3_O)(SO_4_)(C_5_H_5_N)_2_]·0.5C_4_H_10_O
*M* *_r_* = 707.69
Monoclinic, 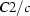

*a* = 26.484 (2) Å
*b* = 14.0374 (15) Å
*c* = 16.8083 (17) Å
β = 108.404 (2)°
*V* = 5929.2 (10) Å^3^
*Z* = 8
Mo *K*α radiation
μ = 1.56 mm^−1^
*T* = 298 K
0.38 × 0.32 × 0.16 mm

#### Data collection

Bruker SMART APEX CCD area-detector diffractometer
Absorption correction: multi-scan (*SADABS*; Sheldrick, 1996 ▶) *T*_min_ = 0.589, *T*_max_ = 0.789
14633 measured reflections
5215 independent reflections
3268 reflections with *I* > 2σ(*I*)
*R*_int_ = 0.055

#### Refinement

*R*[*F* ^2^ > 2σ(*F* ^2^)] = 0.045
*wR*(*F* ^2^) = 0.130
*S* = 1.01
5215 reflections
394 parameters
H-atom parameters constrained
Δρ_max_ = 0.85 e Å^−3^
Δρ_min_ = −0.49 e Å^−3^

### 

Data collection: *SMART* (Siemens, 1996 ▶); cell refinement: *SAINT* (Siemens, 1996 ▶); data reduction: *SAINT*; program(s) used to solve structure: *SHELXS97* (Sheldrick, 2008 ▶); program(s) used to refine structure: *SHELXL97* (Sheldrick, 2008 ▶); molecular graphics: *SHELXTL* (Sheldrick, 2008 ▶); software used to prepare material for publication: *SHELXTL*.

## Supplementary Material

Crystal structure: contains datablocks I, global. DOI: 10.1107/S1600536809012653/ez2166sup1.cif

Structure factors: contains datablocks I. DOI: 10.1107/S1600536809012653/ez2166Isup2.hkl

Additional supplementary materials:  crystallographic information; 3D view; checkCIF report

## Figures and Tables

**Table 1:** Selected bond lengths (Å)

Cu1—O2	1.888 (3)
Cu1—N2	1.957 (4)
Cu1—O1	1.964 (3)
Cu1—N4	2.008 (4)
Cu1—O5^i^	2.389 (3)
Cu2—O3	2.011 (3)
Cu2—N3	2.015 (4)
Cu2—N1	2.016 (4)
Cu2—O4	2.036 (3)
Cu2—N5	2.189 (4)

Symmetry code: (i) 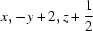.

**Table 2:** Hydrogen-bond geometry (Å, °)

*D*—H⋯*A*	*D*—H	H⋯*A*	*D*⋯*A*	*D*—H⋯*A*
C26—H26⋯O2^i^	0.93	2.46	3.386 (7)	174
C5—H5⋯O3^ii^	0.93	2.43	3.340 (7)	165
C19—H19⋯O7^iii^	0.93	2.46	3.266 (7)	145

Symmetry codes: (i) 
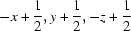
; (ii) 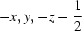; (iii) 
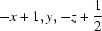
.

**Fig. 1.**
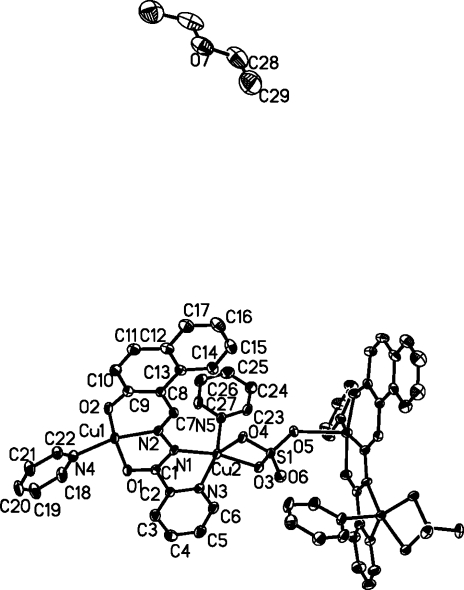
The molecular structure of the compound, showing 30% probability displacement ellipsoids. Unlabelled atoms are related to the labelled ones by symmetry operation (+x, 2 - y, -1/2 + z). H atoms have been omitted for clarity.

**Fig. 2.**
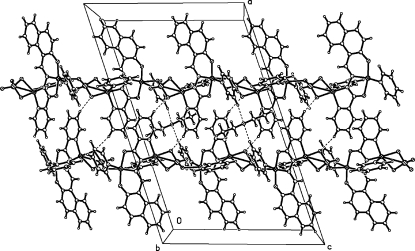
The one-dimensional polymeric structure of the title complex.

## supplementary crystallographic information

### Comment

Hydrazone complexes play an important role in the fields of photoelectric materials and medicines due to their biological and pharmacological activities (Armstrong *et al.*, 2003). The molecular structure of the related salicylaldehyde-2-pyridinecarboxyl-hydrazone has been reported (Bai *et al.*, 2006). To throw further light on the coordination characteristics of 2-pyridinecarboxyl-hydrazone and to explore the properties of their complexes, we report the structure of the title complex (I).

The structure of repeating unit of complex I is shown in Fig. 1 and the one-dimensional polymeric chain structure of the complex is shown in Fig. 2. In the complex, each Cu ion is located in a distorted square pyramidal coordination environment. Cu1 is coordinated to two N and three O atoms, while Cu2 links to two O and three N atoms. The Cu—O (2-naphthol) distance [1.888 (3) Å] is slightly shorter than the previously reported Cu—O (phenol) distance [1.954 (3) Å] (Bai *et al.*, 2006), whereas the Cu—O (carbozone) distance [1.964 (3) Å] is longer than the related Cu—O (carbozone) distance of 1.942 (3) Å in the related complex (Bai *et al.*, 2006). In the crystal, weak intermolecular C—H···O interactions connect the chains into a two-dimensional net structure.

### Experimental

The title compound was synthesizd by mixing 2-hydroxy-1-naphthylaldehyde-2-pyridinecarboxyl-hydrazone (0.0291 g, 0.1 mmol) and copper sulfonate (0.0319 g, 0.2 mmol) and stirring in 10 ml of pyridine for 6 h. The product was filtered and then layered with ether. 2 weeks later brown single crystals were obtained. Anal. Calcd (%) for 2(C27 H21 Cu2 N5 O6 S), C4 H10 O (Mr = 1415.44): C:49.22; H:3.70; N:9.90; Found (%): C: 49.30; H: 3.52; N: 9.76

### Refinement

All H atoms were placed geometrically and treated as riding on their parent atoms, with C—H 0.97 (methylene), C—H 0.93 (pyridine) C—H 0.93 (naphthalene) Å [*U*_iso_(H) = 1.2*U*_eq_(C)], and with C—H 0.96 Å (methyl) [*U*_iso_(H) = 1.5*U*_eq_(C)].

### Crystal data

**Table:**

[Cu_2_(C_17_H_11_N_3_O)(SO_4_)(C_5_H_5_N)_2_]·0.5C_4_H_10_O	*F*(000) = 2888
*M**_r_* = 707.69	*D*_x_ = 1.586 Mg m^−^^3^
Monoclinic, *C*2/*c*	Mo *K*α radiation, λ = 0.71073 Å
Hall symbol: -C 2yc	Cell parameters from 2858 reflections
*a* = 26.484 (2) Å	θ = 2.3–25.3°
*b* = 14.0374 (15) Å	µ = 1.56 mm^−^^1^
*c* = 16.8083 (17) Å	*T* = 298 K
β = 108.404 (2)°	Block, brown
*V* = 5929.2 (10) Å^3^	0.38 × 0.32 × 0.16 mm
*Z* = 8	

### Data collection

**Table:**

Bruker SMART APEX CCD area-detector diffractometer	5215 independent reflections
Radiation source: fine-focus sealed tube	3268 reflections with *I* > 2σ(*I*)
graphite	*R*_int_ = 0.055
phi and ω scans	θ_max_ = 25.0°, θ_min_ = 1.6°
Absorption correction: multi-scan (*SADABS*; Sheldrick, 1996)	*h* = −31→31
*T*_min_ = 0.589, *T*_max_ = 0.789	*k* = −16→16
14633 measured reflections	*l* = −12→19

### Refinement

**Table:**

Refinement on *F*^2^	Primary atom site location: structure-invariant direct methods
Least-squares matrix: full	Secondary atom site location: difference Fourier map
*R*[*F*^2^ > 2σ(*F*^2^)] = 0.045	Hydrogen site location: inferred from neighbouring sites
*wR*(*F*^2^) = 0.130	H-atom parameters constrained
*S* = 1.01	*w* = 1/[σ^2^(*F*_o_^2^) + (0.052*P*)^2^ + 19.1978*P*] where *P* = (*F*_o_^2^ + 2*F*_c_^2^)/3
5215 reflections	(Δ/σ)_max_ = 0.001
394 parameters	Δρ_max_ = 0.85 e Å^−^^3^
0 restraints	Δρ_min_ = −0.49 e Å^−^^3^

### Special details

**Table:**

Geometry. All e.s.d.'s (except the e.s.d. in the dihedral angle between two l.s. planes) are estimated using the full covariance matrix. The cell e.s.d.'s are taken into account individually in the estimation of e.s.d.'s in distances, angles and torsion angles; correlations between e.s.d.'s in cell parameters are only used when they are defined by crystal symmetry. An approximate (isotropic) treatment of cell e.s.d.'s is used for estimating e.s.d.'s involving l.s. planes.
Refinement. Refinement of *F*^2^ against ALL reflections. The weighted *R*-factor *wR* and goodness of fit *S* are based on *F*^2^, conventional *R*-factors *R* are based on *F*, with *F* set to zero for negative *F*^2^. The threshold expression of *F*^2^ > σ(*F*^2^) is used only for calculating *R*-factors(gt) *etc*. and is not relevant to the choice of reflections for refinement. *R*-factors based on *F*^2^ are statistically about twice as large as those based on *F*, and *R*- factors based on ALL data will be even larger.

### Fractional atomic coordinates and isotropic or equivalent isotropic displacement parameters (Å^2^)

**Table:**

	*x*	*y*	*z*	*U*_iso_*/*U*_eq_	
Cu1	0.19607 (2)	0.78202 (4)	0.14330 (4)	0.0327 (2)	
Cu2	0.14395 (2)	1.00409 (4)	−0.08707 (4)	0.03123 (19)	
N1	0.16100 (16)	0.9077 (3)	0.0066 (2)	0.0299 (10)	
N2	0.20801 (16)	0.8671 (3)	0.0587 (3)	0.0297 (10)	
N3	0.06961 (16)	0.9731 (3)	−0.0874 (3)	0.0331 (11)	
N4	0.17578 (17)	0.6701 (3)	0.2012 (3)	0.0347 (11)	
N5	0.16690 (18)	1.1384 (3)	−0.0190 (3)	0.0368 (11)	
O1	0.12165 (13)	0.8167 (3)	0.0856 (2)	0.0366 (9)	
O2	0.26870 (13)	0.7475 (3)	0.1794 (2)	0.0374 (9)	
O3	0.12067 (13)	1.0678 (2)	−0.2000 (2)	0.0355 (9)	
O4	0.20303 (13)	0.9942 (2)	−0.1402 (2)	0.0352 (9)	
O5	0.19783 (14)	1.1264 (3)	−0.2360 (2)	0.0406 (9)	
O6	0.15828 (16)	0.9758 (3)	−0.2900 (2)	0.0449 (10)	
O7	1.0000	0.6854 (6)	0.2500	0.092 (3)	
S1	0.17080 (5)	1.04166 (9)	−0.22080 (8)	0.0293 (3)	
C1	0.1195 (2)	0.8746 (4)	0.0264 (3)	0.0307 (12)	
C2	0.0669 (2)	0.9078 (4)	−0.0292 (3)	0.0326 (12)	
C3	0.0197 (2)	0.8731 (4)	−0.0246 (4)	0.0456 (15)	
H3	0.0191	0.8286	0.0161	0.055*	
C4	−0.0273 (2)	0.9056 (5)	−0.0820 (4)	0.0550 (17)	
H4	−0.0600	0.8828	−0.0808	0.066*	
C5	−0.0247 (2)	0.9719 (5)	−0.1405 (4)	0.0532 (17)	
H5	−0.0557	0.9948	−0.1794	0.064*	
C6	0.0243 (2)	1.0040 (4)	−0.1408 (4)	0.0442 (15)	
H6	0.0257	1.0494	−0.1803	0.053*	
C7	0.2514 (2)	0.8798 (3)	0.0399 (3)	0.0313 (12)	
H7	0.2495	0.9184	−0.0059	0.038*	
C8	0.3020 (2)	0.8388 (3)	0.0846 (3)	0.0297 (12)	
C9	0.3072 (2)	0.7729 (4)	0.1518 (3)	0.0321 (12)	
C10	0.3584 (2)	0.7317 (4)	0.1922 (4)	0.0411 (14)	
H10	0.3616	0.6859	0.2335	0.049*	
C11	0.4018 (2)	0.7567 (4)	0.1727 (4)	0.0439 (15)	
H11	0.4345	0.7299	0.2024	0.053*	
C12	0.3994 (2)	0.8238 (4)	0.1071 (4)	0.0396 (14)	
C13	0.3491 (2)	0.8645 (4)	0.0621 (3)	0.0332 (13)	
C14	0.3484 (2)	0.9312 (4)	−0.0009 (4)	0.0464 (15)	
H14	0.3162	0.9585	−0.0319	0.056*	
C15	0.3939 (3)	0.9568 (5)	−0.0177 (4)	0.0624 (19)	
H15	0.3920	1.0009	−0.0598	0.075*	
C16	0.4427 (3)	0.9177 (5)	0.0272 (5)	0.067 (2)	
H16	0.4734	0.9358	0.0156	0.080*	
C17	0.4451 (2)	0.8525 (5)	0.0886 (4)	0.0556 (18)	
H17	0.4779	0.8265	0.1188	0.067*	
C18	0.1284 (2)	0.6663 (4)	0.2132 (4)	0.0499 (16)	
H18	0.1068	0.7200	0.2012	0.060*	
C19	0.1099 (3)	0.5857 (5)	0.2430 (4)	0.0609 (19)	
H19	0.0768	0.5859	0.2512	0.073*	
C20	0.1414 (3)	0.5058 (5)	0.2601 (4)	0.0580 (18)	
H20	0.1294	0.4502	0.2785	0.070*	
C21	0.1905 (3)	0.5093 (4)	0.2498 (4)	0.0531 (17)	
H21	0.2129	0.4567	0.2628	0.064*	
C22	0.2066 (2)	0.5922 (4)	0.2198 (3)	0.0404 (14)	
H22	0.2399	0.5938	0.2124	0.048*	
C23	0.1707 (2)	1.2197 (4)	−0.0595 (4)	0.0447 (15)	
H23	0.1622	1.2175	−0.1176	0.054*	
C24	0.1862 (3)	1.3053 (4)	−0.0203 (4)	0.0517 (17)	
H24	0.1877	1.3598	−0.0510	0.062*	
C25	0.1995 (3)	1.3085 (5)	0.0656 (4)	0.0590 (18)	
H25	0.2109	1.3654	0.0939	0.071*	
C26	0.1959 (3)	1.2281 (5)	0.1093 (4)	0.0578 (18)	
H26	0.2042	1.2295	0.1673	0.069*	
C27	0.1797 (2)	1.1443 (4)	0.0646 (4)	0.0473 (16)	
H27	0.1776	1.0894	0.0943	0.057*	
C28	0.9808 (4)	0.7425 (7)	0.1773 (8)	0.109 (4)	
H28A	1.0083	0.7862	0.1733	0.131*	
H28B	0.9504	0.7795	0.1798	0.131*	
C29	0.9644 (4)	0.6756 (9)	0.1002 (7)	0.136 (4)	
H29A	0.9954	0.6439	0.0952	0.204*	
H29B	0.9481	0.7122	0.0505	0.204*	
H29C	0.9396	0.6291	0.1072	0.204*	

### Atomic displacement parameters (Å^2^)

**Table:**

	*U*^11^	*U*^22^	*U*^33^	*U*^12^	*U*^13^	*U*^23^
Cu1	0.0341 (4)	0.0334 (4)	0.0307 (4)	0.0030 (3)	0.0104 (3)	0.0091 (3)
Cu2	0.0336 (4)	0.0347 (4)	0.0247 (3)	0.0023 (3)	0.0083 (3)	0.0051 (3)
N1	0.028 (2)	0.034 (2)	0.024 (2)	0.005 (2)	0.002 (2)	0.007 (2)
N2	0.032 (2)	0.028 (2)	0.026 (2)	0.002 (2)	0.004 (2)	0.0031 (19)
N3	0.029 (2)	0.038 (3)	0.029 (2)	0.004 (2)	0.004 (2)	0.001 (2)
N4	0.036 (3)	0.036 (3)	0.033 (3)	0.008 (2)	0.012 (2)	0.005 (2)
N5	0.049 (3)	0.037 (3)	0.025 (2)	−0.004 (2)	0.012 (2)	−0.002 (2)
O1	0.033 (2)	0.044 (2)	0.033 (2)	0.0032 (17)	0.0119 (18)	0.0116 (18)
O2	0.036 (2)	0.043 (2)	0.034 (2)	0.0059 (18)	0.0127 (18)	0.0159 (18)
O3	0.034 (2)	0.044 (2)	0.0280 (19)	0.0051 (17)	0.0087 (17)	0.0052 (17)
O4	0.034 (2)	0.045 (2)	0.0258 (19)	0.0071 (17)	0.0084 (17)	0.0092 (17)
O5	0.050 (2)	0.039 (2)	0.033 (2)	−0.0119 (19)	0.0138 (19)	0.0061 (18)
O6	0.061 (3)	0.044 (2)	0.034 (2)	−0.006 (2)	0.020 (2)	−0.0134 (18)
O7	0.071 (5)	0.065 (5)	0.150 (8)	0.000	0.047 (6)	0.000
S1	0.0337 (7)	0.0319 (7)	0.0224 (7)	−0.0006 (6)	0.0089 (6)	0.0004 (6)
C1	0.033 (3)	0.033 (3)	0.026 (3)	0.000 (2)	0.008 (2)	−0.002 (2)
C2	0.030 (3)	0.037 (3)	0.030 (3)	0.000 (2)	0.009 (2)	0.002 (2)
C3	0.039 (3)	0.053 (4)	0.046 (4)	0.004 (3)	0.016 (3)	0.011 (3)
C4	0.030 (3)	0.071 (5)	0.064 (4)	−0.004 (3)	0.016 (3)	0.006 (4)
C5	0.033 (3)	0.079 (5)	0.044 (4)	0.009 (3)	0.007 (3)	0.010 (4)
C6	0.040 (4)	0.053 (4)	0.036 (3)	0.008 (3)	0.007 (3)	0.010 (3)
C7	0.038 (3)	0.028 (3)	0.029 (3)	−0.003 (2)	0.011 (3)	0.000 (2)
C8	0.032 (3)	0.027 (3)	0.029 (3)	0.000 (2)	0.009 (2)	−0.002 (2)
C9	0.036 (3)	0.029 (3)	0.032 (3)	0.003 (2)	0.012 (3)	−0.002 (2)
C10	0.034 (3)	0.040 (3)	0.045 (3)	0.006 (3)	0.006 (3)	0.008 (3)
C11	0.031 (3)	0.041 (3)	0.055 (4)	0.008 (3)	0.006 (3)	0.004 (3)
C12	0.032 (3)	0.035 (3)	0.054 (4)	0.003 (3)	0.016 (3)	−0.007 (3)
C13	0.031 (3)	0.034 (3)	0.037 (3)	−0.004 (2)	0.015 (3)	−0.007 (3)
C14	0.043 (4)	0.053 (4)	0.047 (4)	−0.005 (3)	0.019 (3)	0.006 (3)
C15	0.065 (5)	0.069 (5)	0.063 (5)	−0.011 (4)	0.034 (4)	0.010 (4)
C16	0.054 (5)	0.071 (5)	0.085 (6)	−0.009 (4)	0.037 (4)	0.003 (4)
C17	0.043 (4)	0.057 (4)	0.070 (5)	0.003 (3)	0.022 (4)	−0.002 (4)
C18	0.050 (4)	0.047 (4)	0.060 (4)	0.021 (3)	0.028 (3)	0.021 (3)
C19	0.052 (4)	0.064 (5)	0.080 (5)	0.008 (4)	0.040 (4)	0.027 (4)
C20	0.059 (4)	0.055 (4)	0.067 (5)	0.004 (4)	0.028 (4)	0.032 (4)
C21	0.057 (4)	0.041 (4)	0.066 (4)	0.011 (3)	0.024 (4)	0.023 (3)
C22	0.043 (3)	0.038 (3)	0.044 (4)	0.007 (3)	0.018 (3)	0.008 (3)
C23	0.060 (4)	0.044 (4)	0.027 (3)	−0.003 (3)	0.010 (3)	0.000 (3)
C24	0.066 (4)	0.036 (3)	0.048 (4)	−0.004 (3)	0.011 (3)	−0.002 (3)
C25	0.068 (5)	0.047 (4)	0.055 (4)	−0.004 (3)	0.010 (4)	−0.016 (3)
C26	0.072 (5)	0.066 (5)	0.030 (3)	−0.006 (4)	0.009 (3)	−0.016 (3)
C27	0.064 (4)	0.047 (4)	0.033 (3)	−0.002 (3)	0.017 (3)	0.007 (3)
C28	0.076 (6)	0.081 (7)	0.184 (12)	0.020 (5)	0.058 (7)	0.039 (7)
C29	0.115 (9)	0.170 (12)	0.139 (10)	0.002 (8)	0.063 (8)	0.014 (10)

### Geometric parameters (Å, °)

**Table:**

Cu1—O2	1.888 (3)	C8—C13	1.459 (7)
Cu1—N2	1.957 (4)	C9—C10	1.432 (7)
Cu1—O1	1.964 (3)	C10—C11	1.339 (7)
Cu1—N4	2.008 (4)	C10—H10	0.9300
Cu1—O5^i^	2.389 (3)	C11—C12	1.436 (8)
Cu2—O3	2.011 (3)	C11—H11	0.9300
Cu2—N3	2.015 (4)	C12—C17	1.402 (8)
Cu2—N1	2.016 (4)	C12—C13	1.426 (7)
Cu2—O4	2.036 (3)	C13—C14	1.409 (8)
Cu2—N5	2.189 (4)	C14—C15	1.368 (8)
N1—C1	1.328 (6)	C14—H14	0.9300
N1—N2	1.398 (5)	C15—C16	1.388 (9)
N2—C7	1.296 (6)	C15—H15	0.9300
N3—C6	1.325 (6)	C16—C17	1.365 (9)
N3—C2	1.359 (6)	C16—H16	0.9300
N4—C18	1.333 (7)	C17—H17	0.9300
N4—C22	1.341 (6)	C18—C19	1.387 (8)
N5—C27	1.340 (7)	C18—H18	0.9300
N5—C23	1.349 (7)	C19—C20	1.372 (8)
O1—C1	1.273 (6)	C19—H19	0.9300
O2—C9	1.297 (6)	C20—C21	1.367 (8)
O3—S1	1.520 (4)	C20—H20	0.9300
O4—S1	1.510 (3)	C21—C22	1.387 (7)
O5—S1	1.452 (4)	C21—H21	0.9300
O5—Cu1^ii^	2.389 (3)	C22—H22	0.9300
O6—S1	1.440 (4)	C23—C24	1.371 (8)
O7—C28^iii^	1.416 (10)	C23—H23	0.9300
O7—C28	1.416 (10)	C24—C25	1.374 (9)
C1—C2	1.486 (7)	C24—H24	0.9300
C2—C3	1.366 (7)	C25—C26	1.366 (9)
C3—C4	1.391 (8)	C25—H25	0.9300
C3—H3	0.9300	C26—C27	1.389 (8)
C4—C5	1.371 (8)	C26—H26	0.9300
C4—H4	0.9300	C27—H27	0.9300
C5—C6	1.376 (8)	C28—C29	1.547 (13)
C5—H5	0.9300	C28—H28A	0.9700
C6—H6	0.9300	C28—H28B	0.9700
C7—C8	1.434 (7)	C29—H29A	0.9600
C7—H7	0.9300	C29—H29B	0.9600
C8—C9	1.433 (7)	C29—H29C	0.9600
			
O2—Cu1—N2	90.44 (16)	C7—C8—C13	120.0 (5)
O2—Cu1—O1	169.76 (15)	O2—C9—C10	117.0 (5)
N2—Cu1—O1	81.79 (16)	O2—C9—C8	124.6 (5)
O2—Cu1—N4	92.59 (16)	C10—C9—C8	118.5 (5)
N2—Cu1—N4	163.41 (17)	C11—C10—C9	122.4 (5)
O1—Cu1—N4	93.03 (16)	C11—C10—H10	118.8
O2—Cu1—O5^i^	96.92 (14)	C9—C10—H10	118.8
N2—Cu1—O5^i^	108.92 (15)	C10—C11—C12	121.8 (5)
O1—Cu1—O5^i^	91.92 (14)	C10—C11—H11	119.1
N4—Cu1—O5^i^	86.91 (16)	C12—C11—H11	119.1
O3—Cu2—N3	94.91 (16)	C17—C12—C13	119.7 (6)
O3—Cu2—N1	164.13 (16)	C17—C12—C11	121.7 (5)
N3—Cu2—N1	80.49 (17)	C13—C12—C11	118.6 (5)
O3—Cu2—O4	70.46 (13)	C14—C13—C12	117.0 (5)
N3—Cu2—O4	150.44 (16)	C14—C13—C8	123.3 (5)
N1—Cu2—O4	106.43 (15)	C12—C13—C8	119.7 (5)
O3—Cu2—N5	93.57 (15)	C15—C14—C13	121.7 (6)
N3—Cu2—N5	107.27 (17)	C15—C14—H14	119.1
N1—Cu2—N5	102.30 (16)	C13—C14—H14	119.1
O4—Cu2—N5	99.37 (16)	C14—C15—C16	120.9 (6)
C1—N1—N2	110.1 (4)	C14—C15—H15	119.6
C1—N1—Cu2	115.5 (3)	C16—C15—H15	119.6
N2—N1—Cu2	134.3 (3)	C17—C16—C15	119.3 (6)
C7—N2—N1	118.2 (4)	C17—C16—H16	120.3
C7—N2—Cu1	128.3 (4)	C15—C16—H16	120.3
N1—N2—Cu1	112.8 (3)	C16—C17—C12	121.4 (6)
C6—N3—C2	117.8 (5)	C16—C17—H17	119.3
C6—N3—Cu2	127.3 (4)	C12—C17—H17	119.3
C2—N3—Cu2	114.7 (3)	N4—C18—C19	123.0 (5)
C18—N4—C22	117.6 (5)	N4—C18—H18	118.5
C18—N4—Cu1	120.9 (4)	C19—C18—H18	118.5
C22—N4—Cu1	121.0 (4)	C20—C19—C18	118.8 (6)
C27—N5—C23	116.2 (5)	C20—C19—H19	120.6
C27—N5—Cu2	122.6 (4)	C18—C19—H19	120.6
C23—N5—Cu2	121.1 (3)	C21—C20—C19	118.8 (6)
C1—O1—Cu1	109.5 (3)	C21—C20—H20	120.6
C9—O2—Cu1	130.7 (3)	C19—C20—H20	120.6
S1—O3—Cu2	94.59 (17)	C20—C21—C22	119.4 (6)
S1—O4—Cu2	93.93 (17)	C20—C21—H21	120.3
S1—O5—Cu1^ii^	135.4 (2)	C22—C21—H21	120.3
C28^iii^—O7—C28	110.9 (11)	N4—C22—C21	122.3 (5)
O6—S1—O5	112.6 (2)	N4—C22—H22	118.8
O6—S1—O4	111.3 (2)	C21—C22—H22	118.8
O5—S1—O4	110.4 (2)	N5—C23—C24	123.9 (5)
O6—S1—O3	110.2 (2)	N5—C23—H23	118.0
O5—S1—O3	111.0 (2)	C24—C23—H23	118.0
O4—S1—O3	100.8 (2)	C23—C24—C25	118.2 (6)
O1—C1—N1	125.7 (5)	C23—C24—H24	120.9
O1—C1—C2	119.5 (5)	C25—C24—H24	120.9
N1—C1—C2	114.8 (5)	C26—C25—C24	120.0 (6)
N3—C2—C3	122.6 (5)	C26—C25—H25	120.0
N3—C2—C1	114.3 (4)	C24—C25—H25	120.0
C3—C2—C1	123.1 (5)	C25—C26—C27	118.1 (6)
C2—C3—C4	118.6 (6)	C25—C26—H26	121.0
C2—C3—H3	120.7	C27—C26—H26	121.0
C4—C3—H3	120.7	N5—C27—C26	123.6 (6)
C5—C4—C3	118.9 (6)	N5—C27—H27	118.2
C5—C4—H4	120.5	C26—C27—H27	118.2
C3—C4—H4	120.5	O7—C28—C29	107.9 (8)
C4—C5—C6	119.1 (6)	O7—C28—H28A	110.1
C4—C5—H5	120.5	C29—C28—H28A	110.1
C6—C5—H5	120.5	O7—C28—H28B	110.1
N3—C6—C5	123.0 (6)	C29—C28—H28B	110.1
N3—C6—H6	118.5	H28A—C28—H28B	108.4
C5—C6—H6	118.5	C28—C29—H29A	109.5
N2—C7—C8	124.8 (5)	C28—C29—H29B	109.5
N2—C7—H7	117.6	H29A—C29—H29B	109.5
C8—C7—H7	117.6	C28—C29—H29C	109.5
C9—C8—C7	120.9 (5)	H29A—C29—H29C	109.5
C9—C8—C13	119.1 (5)	H29B—C29—H29C	109.5

Symmetry codes: (i) *x*, −*y*+2, *z*+1/2; (ii) *x*, −*y*+2, *z*−1/2; (iii) −*x*+2, *y*, −*z*+1/2.

### Hydrogen-bond geometry (Å, °)

**Table:**

*D*—H···*A*	*D*—H	H···*A*	*D*···*A*	*D*—H···*A*
C26—H26···O2^iv^	0.93	2.46	3.386 (7)	174
C5—H5···O3^v^	0.93	2.43	3.340 (7)	165
C19—H19···O7^vi^	0.93	2.46	3.266 (7)	145

Symmetry codes: (iv) −*x*+1/2, *y*+1/2, −*z*+1/2; (v) −*x*, *y*, −*z*−1/2; (vi) −*x*+1, *y*, −*z*+1/2.
